# Stromatolites as Biosignatures of Atmospheric Oxygenation: Carbonate Biomineralization and UV-C Resilience in a *Geitlerinema* sp. *-* Dominated Culture

**DOI:** 10.3389/fmicb.2020.00948

**Published:** 2020-05-19

**Authors:** Rabja M. Popall, Henk Bolhuis, Gerard Muyzer, Mónica Sánchez-Román

**Affiliations:** ^1^Earth Sciences Department, Faculty of Science, Vrije Universiteit, Amsterdam, Netherlands; ^2^Marine Microbiology & Biogeochemistry Department, Royal Netherlands Institute for Sea Research, Utrecht University, Den Hoorn, Netherlands; ^3^Microbial Systems Ecology, Department of Freshwater and Marine Ecology, Institute for Biodiversity and Ecosystem Dynamics, University of Amsterdam, Amsterdam, Netherlands

**Keywords:** cyanobacteria, biomineralization, UV radiation, stromatolite, microbial carbonate, microbial mats, *Geitlerinema* sp., biosignatures

## Abstract

Modern stromatolites are key to the record of past microbial activity preserved in fossil carbonate deposits. Mono-phototrophic cultures dominated by the cyanobacterium *Geitlerinema* sp. were obtained from a laboratory-maintained, low magnesium-calcite stromatolite originating from Lagoa Vermelha, Brazil. This lagoonal system has been described as a Precambrian analog, illustrating a period of photosynthetically induced atmospheric oxygenation, which created a global sanctuary from shortwave solar radiation and enabled the evolution of modern life on Earth. The enrichment cultures precipitate carbonates in minimal media, suggesting that cyanobacterial photosynthesis and extracellular polymeric substance production may be crucial in the mineralization of the studied stromatolite. We further show that *Geitlerinema* sp. can build and maintain filamentous mats under long-term UV-C exposure. Our results suggest that present day stromatolites dominated by cyanobacteria may be interpreted as biosignatures of atmospheric oxygenation and have implications for the search for putative biological traces on Mars.

## Introduction

Stromatolites are the oldest known fossil records of life on Earth. The organo-sedimentary structures are formed by complex interactions between microbial mat communities and their geochemical environment, thus providing insight into the ecosystem at the time of their genesis to approximately 3.5 Ga ago in the early Archean (Vasconcelos and McKenzie, 2009; Vasconcelos et al., 2014). Stromatolite abundance peaked in the Proterozoic and strongly decreased toward the Cambrian. This decline is attributed to the occurrence of metazoan grazers that started to put trophic pressure on microbial mats (Awramik, 1971). Next to predation, a decrease in sea water carbonate saturation as well as increasingly complex ecosystems with niche diversification and eukaryotic competition are considered to have impacted stromatolite abundance after the Mesoproterozoic (Monty, 1973; Grotzinger, 1990; Riding, 2006). The rare modern stromatolites feature active microbial mat communities forming lithified discrete buildups, and can only be found in few natural environments (Foster et al., 2009; Suosaari et al., 2016). Those habitats are often characterized by an elevated salinity sheltering biofilms from animal grazing and continuous sediment disturbance (Douglas and Beveridge, 1998; Schieber, 2007). In accordance with their shallow aquatic environment, most of the extant stromatolites contain calcareous compounds. They are of great importance to the interpretation of ancient stromatolites and key to their record of past microbial activity forming lithified deposits (Decho and Kawaguchi, 2003; Vasconcelos et al., 2006; Vasconcelos and McKenzie, 2009; Kaźmierczak et al., 2015).

The functionally and structurally diversified modern stromatolites feature comprehensive communities in distinct layers (Vasconcelos and McKenzie, 2009). Comprising complete cycles of C, N, and S, microbial mats form a nearly closed minimal ecosystem and include the major functional groups of oxygenic photosynthetic primary producers (Cyanobacteria), aerobic heterotrophs, sulfur oxidizers, and anaerobic sulfate reducers along a vertical O_2_ gradient (Visscher and Stolz, 2005). By definition, stromatolite growth is promoted “through accretion of laminae by the entrapment of sediment and by participation of carbonate, under active secretion or direct influence of microorganisms” (Altermann, 2008). The mechanism of trapping and binding sediment particles in the microbial mat is less significant in ancient carbonate stromatolites due to the lack of detritus from higher life forms in the Precambrian complementing carbonate sources of micrite and erosion, although some Archean microorganisms secreted envelopes of biopolymers which might be identical to extracellular polymeric substances (EPS) incorporating sedimentary material in present day biofilms (Kazmierczak, 2002). More relevant for such early stromatolites, however, is the biomineralization of calcite, aragonite, and dolomite by the microbial community (Kaźmierczak et al., 2015). Modern environments characterized by an increased salinity and absence of eukaryotic grazers and bioturbators (Vasconcelos et al., 2006), as well as alkaline lake (Kazmierczak and Kempe, 2006) and hot spring (Konhauser et al., 2001; Phoenix et al., 2006) stromatolites, likewise promote autochthonous input of material and are thus considered good textural analogs to Precambrian systems. In contrast, coarsely laminated structures such as Bahamian and Shark Bay stromatolites feature a relevant ratio of allochthonous grains in addition to *in situ* precipitation (Fairchild, 1991). The accumulation of autochthonous carbonates in a mat is the net result of precipitation and counterbalancing dissolution preceding diagenesis and amalgamation with the lithified stromatolite deposit (Visscher and Stolz, 2005). Carbonate precipitation is interpreted as a byproduct of photosynthetic and sulfate reducing metabolism promoting biomineral supersaturation in alkaline microenvironments (Visscher et al., 1998; Kaźmierczak et al., 2015), but only possible on suitable nucleation sites further reducing kinetic barriers (Kamennaya et al., 2012). An ideal matrix for crystal nucleation are the amphiphilic EPS, which feature a high uronic acid content and are suitable for binding both Mg/Ca cations and carbonate anions (Decho and Kawaguchi, 2003; Dittrich and Sibler, 2010). Top-layer cyanobacteria are not only photosynthetically active, but also the primary EPS producers of the system (Decho and Kawaguchi, 2003). This renders them key players contributing majorly to carbonate deposition in modern lithifying mats.

Molecular traces in the lithified stratum of previously studied stromatolites also confirm a significant contribution of sulfate reduction to lamina formation (Vasconcelos et al., 2014). A prominent sulfur cycle, however, was only established as a result of emerging oxygenic photosynthesis during the Great Oxygenation Event (GOE) (Canfield and Raiswell, 1999). While anaerobic photosynthesizers were most likely relevant for the formation of pre-GOE stromatolites (Bosak et al., 2007), the massive proliferation of oxygenic cyanobacteria facilitated a tremendous diversification of life and an increasingly oxygenated atmosphere (Shestakov and Karbysheva, 2017). During the simultaneous establishment of an atmospheric ozone shield, light-dependent cyanobacteria were exposed to the extremely damaging shortwave spectrum of ultraviolet (UV) solar radiation (Garcia-Pichel, 1998). Sedimentary habitats may have additionally aggravated the damaging effect of UV-C due to light-trapping effects, resulting in cyanobacterial mats developing highly effective adaptions such as chemical protectants, DNA repair mechanisms and phototaxis, a behavioral mechanism preserved in fossil stromatolites (Garcia-Pichel, 1998). The role of UV-C resilient cyanobacteria in the evolution of aerobic modern life-forms is not only of paleontological interest, but also significant to understand a possible existence of life on Mars. The finding of organic carbon on Mars recently sparked a new discussion on potential traces of past Martian biota (Eigenbrode et al., 2018). Present surface conditions on Mars are comparable to the ones of early Earth, neither providing an aerobic environment nor protection from UV-C wavelengths (Cockell, 2000). A stromatolitic record of oxygenic cyanobacterial photosynthesis may therefore be key to terraforming approaches as well.

Here, we studied a natural stromatolite maintained under controlled laboratory conditions originating from Lagoa Vermelha, Brazil. The formation of magnesium calcite and dolomite deposits in Lagoa Vermelha is the result of a unique biochemical environment considered to represent the shallow marine systems of early Earth (Vasconcelos et al., 2006). Bordering the South Atlantic, Lagoa Vermelha is part of a large lagoonal site. The proximity of an Atlantic upwelling zone accounts for semi-arid conditions, while periodical changes in surface area and water chemistry are enforced by a mean depth of 2 m (Vasconcelos et al., 2006; Spadafora et al., 2010). The microbial mats of Lagoa Vermelha and other modern stromatolites are essential to understand the biotic mechanisms of carbonate deposition in past systems. In consideration of this relationship, we analyze the microbial community composition by 16S rRNA gene amplicon sequencing and carbonate deposition using stable C and O isotope analysis. Additional experiments were performed with *Geitlerinema* sp. enrichments to test their ability to mineralize carbonate and grow under long-term UV-C radiation.

## Materials and Methods

### Origin and Sampling of the Studied Stromatolite

**FIGURE 1:**
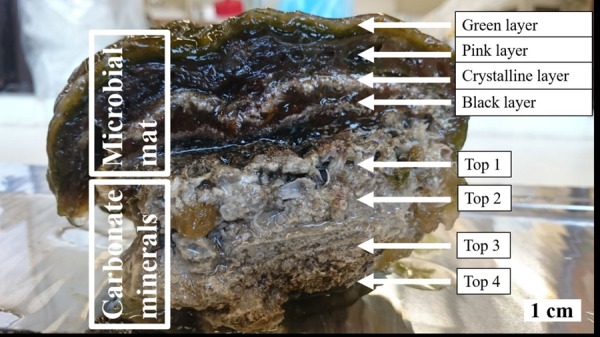
Cross section of the studied stromatolite illustrating the layered microbial mat, which embeds the crystalline layer of unconsolidated precipitates, and relevant zones of the carbonate deposit below. The top green layer generally features photosynthetic cyanobacteria producing primary OM to be consumed by aerobic heterotrophic bacteria in the uppermost part of the layer below. The diverse community of this zone moreover comprises autotrophs, especially the purple sulfur bacteria which give the layer its characteristic pink color and are capable of microaerophilic or anoxygenic photosynthesis in the deeper parts of the zone. Below the distinct layer of unconsolidated precipitates composed of carbonate minerals such as magnesium calcite and/or dolomite, the community is dominated by strictly anoxic conditions and salvages (in-)organic metabolic products of the upper layers in association with less pronounced crystal deposits. The most prominent process in this black layer is sulfate reduction, next to obligate fermentation and other anoxygenic heterotrophy. The carbonate deposit in the lower half of the stromatolite comprises granulated as well as more compact strata toward the bottom.

The studied stromatolite has been kept at room temperature in a hypersaline (>7% salt) aquarium at ETH Zurich, Switzerland, and currently at the Vrije Universiteit in Amsterdam, the Netherlands, for a total period of 15 years. An approximately 0.5 cm deep, 3 cm tall and 7 cm wide slice of the microbial mat was cut from the stromatolite (Figure 1). The biofilm covering the top layer was carefully removed to avoid potential contamination with non-associated algae. The slice was subdivided into smaller pieces according to the intended use and stored in saline solution if not immediately processed. An initial assessment of the nature of the phototrophic community was performed by fluorescence microscopy to distinguish between Chlorophyll *a* containing micro-eukaryotic alga and phycoerythrin containing cyanobacteria. Chlorophyll *a* was visualized using the Carl Zeiss Filter Set 09 (excitation 450–490 nm, emission 515 nm) and phycoerythrin was visualized using the Carl Zeiss Filter Set 20 (excitation 546 nm, emission 575–640 nm).

### Community Composition of Microbial Mat Layers

The microbial mat was dissected into four distinctive layers: The green top layer, the pink layer below, the crystalline layer, and the black bottom layer (Figure 1). All outer parts that were exposed to the external medium were thinly sliced off to avoid contamination. DNA was extracted from each layer using the MoBio PowerSoil DNA Isolation Kit with intensive bead beating for three cycles of 10 s beating at 3.5 m/s intensity and 40 s pause using the Omni Ruptor 24 Elite bead mill homogenizer (Omni Int., United States). 16S rRNA library preparation and Illumina sequencing were subsequently performed at BGI, China using bacterial (341F: ACTCCTACGGGAGGCAGCAG & 806R: GGACTACHVGGGTWTCTAAT) and archaeal (Arch349F: GYGCASCAGKCGMGAAW & Arch806R: GGACTACVSGGGTATCTAAT) specific 16S rRNA primers amplifying the V3-V4 region. Archaeal and bacterial sequences were clustered at a 95% sequence identify cutoff following the recommendations by Cardoso et al. (2017) and annotated following the QIIME version 1 pipeline (Caporaso et al., 2010) using the SILVA version 132 reference database (Quast et al., 2013). OTU abundances were normalized to relative abundance in percentage. The sequencing data is deposited in the NCBI small read archive and listed under BioProject ID PRJNA610984.

### Unconsolidated and Lithified Carbonates

The morphology of unconsolidated precipitates was analyzed via scanning electron microscopy (SEM), while the chemical elemental composition was assessed with energy dispersive X-ray spectroscopy (EDS). Samples from the crystalline layer (Figure 1) were rinsed with ultrapure water and dried for 2 days at room temperature (RT). To remove halite residues, samples for later SEM runs were soaked in ultrapure water overnight. Additionally, the material was washed in ultrapure water and centrifuged at 10,000 × *g*, which was repeated five times. The final pellet was dried at RT. Microscopical analyses were conducted on a FEI Helios Nanolab G3 FIB-SEM. The samples were previewed under an optical microscope, fixed on mounts and coated with a platinum film of 5 nm thickness, before being observed in secondary electron mode at an accelerating voltage of HV = 10 kV and an emission current of 6.3 pA. Both unconsolidated precipitates and lithified deposit were further studied for C and O stable isotopes. The crystalline layer as well as four porous to compact laminae of the carbonate deposit ranked from top to bottom (Figure 1) were sampled, soaked in ultrapure water for 2 days and subsequently dried in an oven at 40°C. Isotope δ^13^C and δ^18^O values were established in relation to the Vienna Pee Dee Belemnite (VPDB) standard on a Finnigan MAT253 mass spectrometer using the Gasbench II. Sample size was corrected using the Vrije Universiteit Amsterdam in house carbonate standard (VICS), while the international IAEA-603 was measured as a control standard. The long-term standard deviation of the routinely analyzed in-house standard is <0.1‰ (1σ) for both carbon and oxygen isotope ratios. Isotopic values were established in four samples per layer.

### Carbonate Biomineralization in a Cyanobacterium-Dominated Culture

#### Cultivation in Solid and Liquid Medium Under Promotion of Carbonate Biomineralization

A sample of the microbial mat was horizontally cut along the crystalline layer. The upper half consisting of green and pink layers was aerobically incubated in room-tempered sterile hypersaline solution under natural lighting. After 7 days, the culture was inoculated to ASN-III-CS growth medium plates (*n* = 15). The original ASN-III medium composition after Rippka et al. (1979) was modified to promote calcium/magnesium carbonate biomineralization and optimized over the course of the study. Additionally, the preparation procedure was adapted to avoid abiotic calcium carbonate precipitation (Supplementary Table S1). The cultures were incubated at 30°C and subjected to artificial illumination (40 W LED at 40 cm distance) in a 12 h light/12 h dark rhythm. After 10 weeks, each plate was individually wrapped in a translucent plastic bag to avoid agar desiccation. The plastic bags were punctured with a needle to allow gas exchange. Three weeks post-observation of cyanobacterial cultures, liquid ASN-III-CL medium (Supplementary Table S1) was inoculated with filamentous cell material from the agar cultures (*n* = 3). A sterile microscope slide was added to provide a retrievable surface for cellular growth and carbonate nucleation. The liquid cultures were incubated aerobically at 30°C and exposed to artificial illumination (40 W LED at 40 cm distance) in a 12 h light/12 h dark rhythm. Cell material was scraped from ASN-III-CS agar grown cultures for molecular analysis. DNA extraction, 16S amplicon sequencing and taxonomic assignment were performed as described above.

#### Identification of Carbonate Precipitates

Crystal nucleation and maturation were monitored with an optical microscope, while established carbonate crusts were analyzed via SEM-EDS. Cell material and carbonate crust were scraped off the agar in ASN-III-CS cultures and centrifuged for 90 s at 10,000 × *g*. The pellet was washed in ultrapure water and centrifuged at 10,000 × *g*, which was repeated three times. The pellet was then dried at RT. The material was put on mounts and coated with platinum as described above. Carbonate crusts from solid ASN-III-CS and organic material from liquid ASN-III-CL culture were furthermore assessed with transmission electron microscopy (TEM), EDS and electron diffraction (ED). One milliliter of cellular material settled on the microscope slide was sampled from liquid cultures and centrifuged for 10 min at 8,000 × *g* according to the preparation protocol in Benzerara et al. (2014). Subsequently, the pellet was washed with ultrapure water by centrifuging three times at 8,000 × *g* and resuspended in 200 μl of ultrapure water. All samples were treated ultra-sonically in a bath sonicator for 60 s. 5 μl of dispersion were pipetted on a glow discharged TEM-grid for analysis on a FEI Talos F200X TEM. Material from solid cultures was examined in TEM bright field mode, while liquid cultures were additionally analyzed in STEM dark field mode and tilted between 30° and 60° if applicable. EDS was conducted to assess the elemental composition of sample compounds and ED to assign a crystalline or amorphous character. In addition, carbonate crusts from solid cultures were subjected to stable isotope analysis. Sample preparation and determination of δ^13^C and δ^18^O values was performed as described above.

### Cyanobacterial Growth Under UV-C Radiation Stress

Free-floating cell material from liquid ASN-III-CL cultures was transferred to sterile saline solution which was used as inoculum for glass beakers containing solid ASN-III-US or liquid ASN-III-UL medium (Supplementary Table S1). The beakers were covered in a plastic wrap punctured with a fine needle to allow gas exchange. A sterile microscope slide was added to the liquid cultures as retrievable surface for cellular growth. Both solid (*n* = 5) and liquid (*n* = 5) cultures plus a negative control without bacterial cells each were placed in a laminar flow chamber beneath a UV-C tube (15 W, peak at 250 nm) mounted at 40 cm distance from the surface of the culture medium. The cultures were subjected to 12 h of both continuous UV-C and photosynthetically active radiation (PAR) combined (40 W LED, also at 40 cm distance), followed by 12 h of darkness, for a total period of 6 weeks. To avoid natural light exposure throughout the day, the chamber was completely covered in tinfoil. A control series, consisting of five solid and five liquid cultures with cells and one solid and one liquid culture without bacterial cells, was placed in an opaque box and exposed to PAR exclusively for 12 h light/12 h dark. Four weeks after the start of the experiment, one of the positive agar controls was added to the UV-C exposed experiment to monitor UVR effect on intact cyanobacterial mats. In response to the appearance of red precipitate in the liquid UVR-exposed experiments, negative controls of ASN-III-UL medium and sterile demineralized water covered in non-punctured plastic wrap were added to examine external contamination. Samples of red precipitate from a culture experiment were centrifuged at 10,000 × *g* for 90 s. The pellet was washed three times in ultrapure water and resuspended in 200 μl. The material was further prepared for TEM-EDS as described above before being examined in TEM bright field mode for imaging and with an EDS detector for elemental analysis. Cyanobacterial mat cover was monitored over a period of 6 weeks in all cultures. Organic material (OM) was prepared for and assessed via TEM-EDS and ED as described above.

## Results

### Characterization of the Laboratory-Incubated Stromatolite

#### Microbial Community Composition of Mat Layers

**FIGURE 2:**
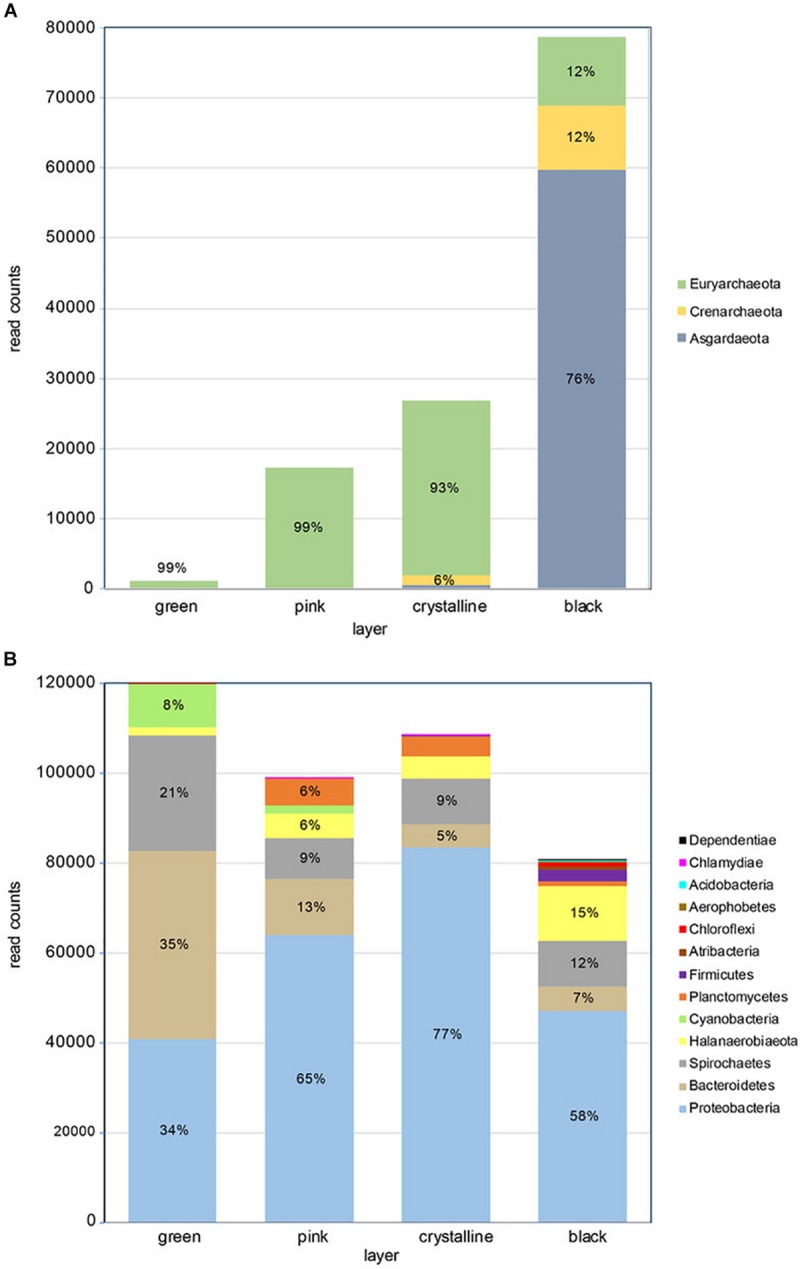
Taxonomic composition of microbial mat layers showing **(A)** archaeal read counts and **(B)** bacterial read counts. The relative phyla frequency within a layer is indicated in percent if respective read counts constitute >5%.

Amplicon sequence analysis of the archaeal and bacteria 16S rRNA V3-V4 region revealed more bacterial than archaeal reads (Figure 2 and Supplementary Figure S1). While the number of archaeal reads increased strongly from the green top layer to the black bottom layer, the bacterial reads showed a reverse tendency with most reads obtained in the top layer and least in the bottom layer. Archaeal diversity was overall low with an estimated Chao-1 richness ranging from 6 in the top layer to 17.5 in the third, crystalline layer. Shannon diversity for the archaeal fraction was highest in the crystalline l (*H* = 1.03) and lowest black layer (*H* = 1.07) (Supplementary Table S2). Evenness was overall low with the two dominant archaeal OTUs, OTU_17 and OTU_29 making up 78% of the total community abundance. The Crystal layer is dominated by OTU_17 and OTU_13, assigned to the orders Methanosarcinales and Halobacteriales, respectively, and the black layer is dominated by OTU_29 and OTU_25, assigned to the classes Lokiarchaeia and Bathyarchaeia.

Diversity estimators for the bacterial community revealed a slight increase in Chao-1 richness from 96 to 135 from the top to the bottom layer (Supplementary Table S2). Shannon diversity index varied little between the three top layers and was highest in the bottom layer (*H* = 3.22). The evenness was overall low (0.13–0.20) indicative for a relative low number of dominant species. Indeed the 14 most abundant OTUs make up more than 75% of the total abundance.

Taxonomic annotation of the bacterial and archaeal OTUs was performed with QIIME using the SILVA version 132 reference database. This provides the taxonomic identifier of the best hits but does not give the percent identity to these hits. The OTUs were therefore also analyzed using standard online NCBI blastn algorithms. This revealed that the majority of the archaeal OTUs gave good hits (mean 98% identity) with nucleotide database (nt), but a very low identity (mean 86%) when comparing to the reference type-strain database (Supplementary Table S3).

The archaeal community in the top three layers mainly consists of Euryarchaeota (>90% of total abundance). These layers are dominated by methanogens of the genus *Methanohalophilus* and to a lesser extent of Halobacteria. The Halobacteria contribution is highest in the third layer (∼13%) but they form less than 0.3% of the archaeal population in the bottom layer. The black bottom layer is dominated by phylum *Asgardarchaeota*, class Lokiarchaeota (76% of total) that are neglectable in the other layers. The bottom layer furthermore contains uncultured members of the orders Bathyarchaeia (∼12%) and Thermoplasmata (∼11% - Marine Benthic Group D).

The bacterial community in the stromatolite is dominated by *Proteobacteria*, with a relative abundance increasing from 34% in the top layer to 77% in the third layer and decreasing to 58% in the lower layer (Figure 2), Bacteroidetes is the dominant phylum in the top layer (35%) and takes the second and third place in dominance in the subsequent layers. Other abundant phyla are *Spirochaetes* (13% of total bacterial community and present in all layers), *Halanaerobiaeota* (6% of total bacterial community and mainly present in the lower layers). *Cyanobacteria* are only significantly present in the top two layers (respectively, 8% and 2%) (Supplementary Table S4). Several genera and families were found that currently have no cultured representatives and no names could be provide but have the prefix _f or _g to indicate unknown family or genus, respectively. Two genera are dominantly found in all layers, *Marinospirillum* and *Marinobacter*, which make up 21% to 30% and 18% to 37%, respectively, in the lower three layers. In the top green layer, they are at a third and fourth place in relative abundance following an uncultured Balneolaceae genus (34%) and uncultured Spirochaeta 2 genus (21%). The top layer is furthermore characterized by the presence of the cyanobacterium *Geitlerinema* (6%). The lower layer is characterized by a relative higher abundance of potential sulfate reducing bacteria (*Desulfosalsimonas*, a novel Desulfobacteraceae genus and *Desulfovibrio*). Sequences derived from potential sulfur oxidizing bacteria were mainly found in the lowest black layer at 0.5% abundance and were annotated as Thiohalospira, a halophilic, obligately chemolithoautotrophic sulfur-oxidizing genus. Among these abundant genera we can recognize members that increase or decrease in abundance with depth of the layers and those that are more abundant in the two middle layers (Supplementary Table S4). A decrease in abundance from top to bottom is especially observed for members of the Balneolaceae_g, Spirochaeta 2, *Geitlerinema*, *Salinarimonas*. Genera that increase in abundance from top to bottom are more numerous and consist of amongst others *Marinospirillum*, *Halanaerobium*, *Sediminispirochaeta*, Oligoflexales_f_g and Marinilabiliaceae_g. The genus *Marinobacter* is especially abundant in the two middle layers. Finally, we identified genera which nearly exclusively reside in one of the four layers with an on average 50 times higher abundance than the sum of the other three layers or were absent in the other layers. Notably were Oligoflexales_f_g, Clostridiales_f_g and Desulfobacteraceae_g in the bottom layer, *Cryomorpha* in the top layer, *Defluviicoccus* in the second and *Pelagibius* in the third layer (Supplementary Table S5).

#### Unconsolidated and Lithified Carbonates

**FIGURE 3:**
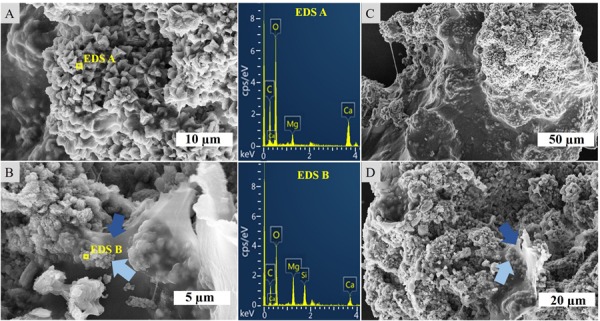
SEM-EDS of the crystalline layer embedded in the stromatolitic microbial mat. **(A)** Imaging and EDS of larger, crystalline structures, **(B)** EDS of spheroids (light blue arrows) adhered to sheaths of EPS (dark blue arrows), **(C)** overview of a carbonate complex, and **(D)** granulated texture of crystalline microstructures and EPS.

SEM-EDS analysis of the crystalline, third layer from the top identified the unconsolidated precipitates as magnesium calcite. The plate-shaped microcrystals (Figure 3A) measured 2–3 μm in length and occurred in aggregates of needle and cauliflower morphology. Aggregates were closely associated with each other, forming textures appearing poorly crystallized (Figures 3C,D). Spheroids below 1 μm in diameter were observed in association with layers of organic material (Figures 3B,D). The OM was layered, contained high rations of C and O and was, thus, related to EPS. All crystalline structures peaked in the C, Ca, and Mg spectra, while spheroids infrequently contained Si in addition (Figures 3A,B). The ratio of stable ^12^C and ^13^C isotopes varied between the crystalline layer and throughout the laminae of the carbonate deposit, averaging around a δ^13^C value of −1.34 in relation to Vienna PeeDee Belemnite (VPDB). The δ^13^C of the unconsolidated precipitates was most negative with a mean value of −2.33‰, with the value rising above zero in the first layer of the deposit, dropping slightly below zero in the porous layer below and sharply decreasing to mean isotopic ratios of −2.16‰ and −2.02‰, respectively, in the compact laminated and bottom layers of the deposit (Table 1). The delta values of stable ^16^O and ^18^O were relatively constant throughout the layers, fluctuating between 0.39‰ and 0.86‰ VPDB, respectively (Table 1).

**TABLE 1:** Mean isotope delta values throughout the layer of crystalline precipitates in the mat (Top 0) and the primary (Top 1), porous (Top 2), compact (Top 3), and bottom (Top 4) layer of the carbonate deposit.

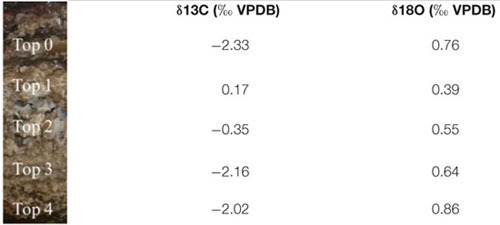

*All total of four samples was measured per layer. The long-term standard deviation of the routinely analyzed in-house standard is <0.1‰ (1σ).*

### Carbonate Biomineralization in Cyanobacteria-Dominated Culture

#### Growth and Taxonomic Composition of Cultures

Fifteen cyanobacterial enrichments were obtained through plating on ASN-III-CS agar and three enrichments through transferring cyanobacterial colonies from ASN-III-CS plates to liquid ASN-III-CL medium. The cyanobacteria formed dense, filamentous mats on agar (Supplementary Figure S2). In liquid medium, cellular growth was monitored on available surfaces (culture vessel, microscope slide) first, while more mature filaments conglomerated in floating mats. The ASN-III-CL culture medium retained a clear color at all times, indicating the strong tendency of cells to associate with each other (Supplementary Figure S3). After 8 weeks of incubation, the bacterial cover started to die off on the increasingly desiccated agar base, while liquid cultures were maintained.

**FIGURE 4:**
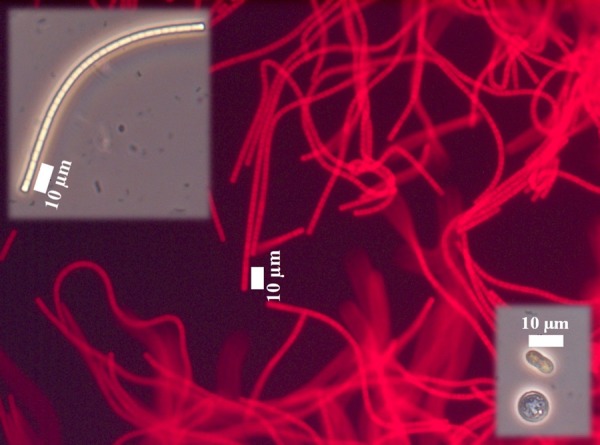
Morphology of isolates from mixed agar culture under an optical microscope in standard adjustment (smaller pictures) and equipped with a green filter (big picture). The EPS appears as white to yellowish sheaths around the cyanobacterial cells.

Both light- and dark-green mats showed similar morphological characteristics. While few coccoid cells of approximately 10 μm diameter could be observed, the vast majority of isolates appeared as flat-topped sheathed filaments of a variety of lengths. The single trichomes consisted of aligning rectangular cells about 2.7 μm in length and 1 μm in height (Figure 4). The majority of cultured cells showed strong red fluorescence when excited at 546 nm and not when excited at 450–490 nm, confirming the presence of phycoerythrin pigmentation characteristic for cyanobacteria (Figure 4). Molecular analysis identified the cyanobacterium as *Geitlerinema* sp. and the cultures as non-axenic containing also members of *Bacteroidetes* (averaging 15%) and *Proteobacteria* (averaging 12%). Since molecular analysis identified no other cyanobacteria in the enrichment cultures, the cultures are mono-phototrophic.

#### Characterization of Culture Carbonate Mineral Precipitates

**FIGURE 5:**
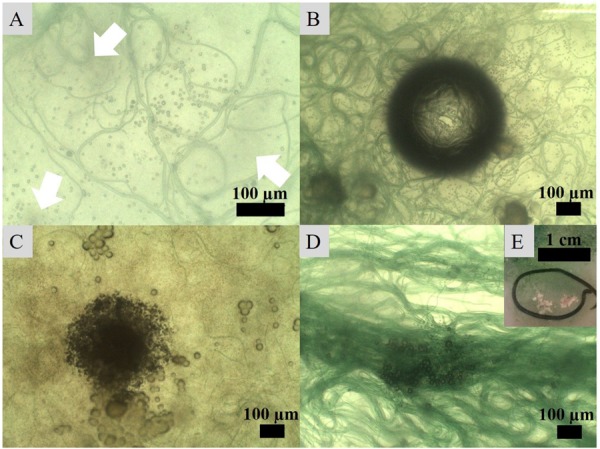
Crystal nucleation and growth observed under an optical microscope. **(A)** Nucleation of globules in association with bacterial filaments and development of darker spots (indicated with arrows) in the medium, **(B)** increased number of nucleating crystals around filaments attracted to a CO_2_ or O_2_ bubble in the medium, **(C)** amalgamation of globules into granulated textures growing into **(D)** larger crystals on the surface of filament clusters and eventually **(E)** into carbonate crusts visible to the naked eye.

**FIGURE 6:**
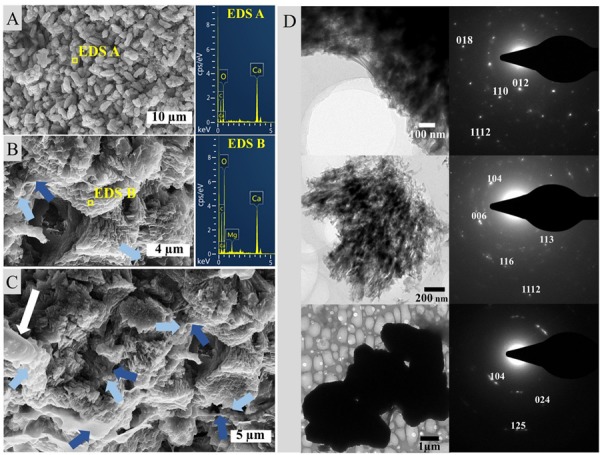
SEM imaging and EDS analysis of carbonate crusts from agar cultures illustrating **(A)** needle morphology, **(B)** aggregate texture including nucleating spheroids (light blue arrows) in EPS sheaths (dark blue arrows) and **(C)** sheaths of EPS with nucleating spheroids as well as a calcified cell (white arrow) associated with spheroids on the far left, and **(D)** indexed ED patterns of calcite sample compounds.

Incubation of the *Geitlerinema* enrichment revealed nucleation of micro-globules after 17 days of incubation on seven agar plates. The dark spheres were approximately 1 μm in diameter and associated with filaments. In close proximity to many of the nucleation sites with denser filament coverage, the agar adopted cloudy spots of darker discoloration (Figures 5A,B). Over the course of the following weeks, the spheroids amalgamated in granulated textures and continued to grow in diameter (Figures 5C,D). Sixty days post-inoculation, white crusts visible to the naked eye and morphologically different from halite appeared on top of bacterial mats (Figure 5E). SEM-EDS analysis of this crust revealed magnesium calcite crystals. Imaging disclosed plate-shaped microstructures (Figure 6A) conglomerated in needle and cauliflower aggregates, which were in turn associated in granulated textures (Figures 6B,C). Additionally, spheroids below 1 μm in diameter could be monitored adhering to EPS layers (Figures 6B,C). EDS spectra of all morphology types revealed constantly high Ca and C peaks, while traces of Mg could be found sporadically (Figures 6A,B). A crystalline character was confirmed via electron diffraction pattern by TEM analysis (Figure 6D). ED patterns were composed of few distinct rings and scattered spots of varying intensities, which were indexed corresponding to the d-spacing values of calcite after Downs et al. (1993). Furthermore, completely calcified cells were disclosed via imaging and chemical elemental analysis (Figure 6C). The mean δ^18^O of carbonate crusts resulted in 0.39‰ VPDB, while the average isotopic ratio of stable carbon isotopes had a value of −12.26‰ VPDB.

**FIGURE 7:**
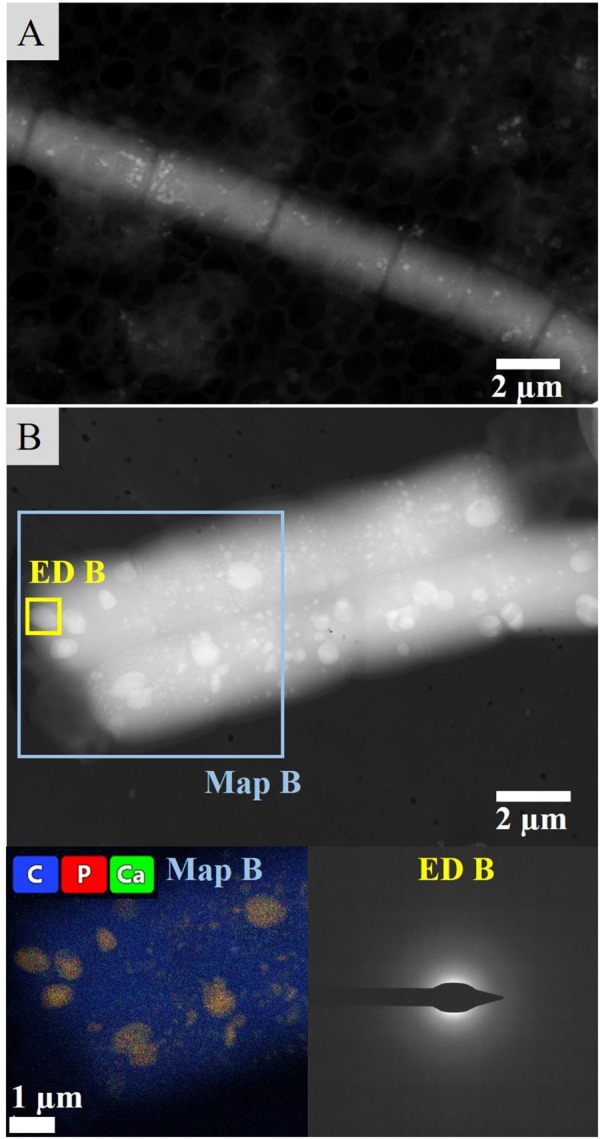
Intracellular inclusions from liquid culture experiments. **(A)** Distribution of inclusions within the cells and throughout a filament visualized in HAADF mode. **(B)** Map and exemplary ED of intracellular granules.

Neither extracellular crystals nor crusts could be observed in liquid cultures. TEM imaging revealed approximately round, intracellular inclusions of a range of diameters below 0.5 μm that were scattered irregularly within the cells and over the length of the filament (Figure 7A). The inclusions were often associated with each other, stayed in place upon tilting of the sample and featured similar atomic weights in HAADF-mode. EDS spectra revealed a high P ratio and lower Ca content, as well as elemental Mg, S and sometimes K peaks above the noise. A crystalline character could not be determined via TEM-ED (Figure 7B).

### Cyanobacteria-Dominated Culture Growth Under UV-C Radiation Stress

**FIGURE 8:**
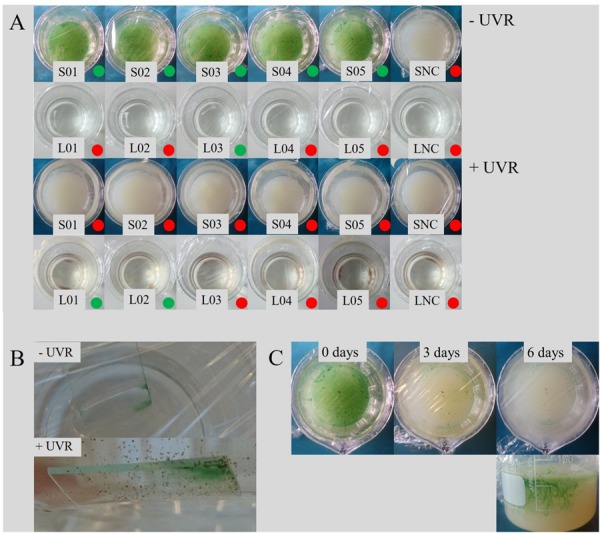
Effects of UVR on cyanobacterial growth. **(A)** Growth in control series and UVR-exposed experiments after 6 weeks, distinguished in solid (S) and liquid (L) cultures including negative controls (NC). Cultures featuring bacterial growth are marked with a green dot, continuously sterile cultures with a red dot. In all liquid cultures subjected to UVR, including the NC, precipitation of red flakes could be observed (see also **B**). **(B)** Cyanobacterial growth occurred exclusively beneath the microscope slide in liquid culture subjected to UV-C, and on only top of the slide in the control. **(C)** Development of an intact cyanobacterial mat in agar culture after exposure to UVR.

**FIGURE 9:**
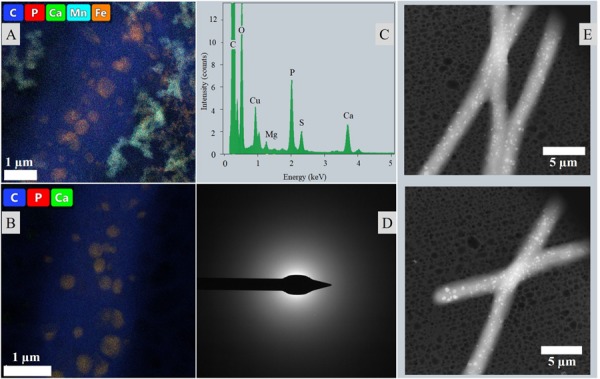
Intracellular inclusions from the UVR experiment. **(A)** Map of a filament from liquid culture exposed to UVR surrounded by the Fe and Mn rich precipitated flakes. **(B)** Map of a filament from an agar control culture. **(C)** Representative EDS of the intracellular inclusions. **(D)** Representative ED of the intracellular inclusions. **(E)** Distribution of inclusions throughout the filaments and cells visualized in HAADF mode.

Liquid cyanobacterial cultures exposed to UV-C radiation revealed the formation of red flakes within a week after the start of the experiment (Figures 8B, 9A). The same phenomenon appeared in all ASN-III-UL negative control cultures (− cells) exposed to UV-C, both covered with punctured and non-punctured plastic wrap, but not in sterile demineralized water. EDS analysis of the flakes revealed high peaks in Fe and Mn (Figure 9A). While all ASN-III-US positive controls (+ cells, – UVC) were covered in a dense cyanobacterial mat within 1 month, no solid cultures could be monitored under UVR exposure (Figure 8A). After 6 weeks, cyanobacterial growth could be observed in two liquid UV-C exposed cultures, albeit exclusively beneath the added microscope slides. In liquid controls without UV-C, cyanobacterial growth was observed in one culture only, and exclusively on the top side of the microscope slide (Figures 8A,B). When subjected to UV-C, the surface bacterial cover of a positive agar control significantly decreased within days and completely diminished after a week. Cyanobacterial filaments embedded in the subsurface agar (∼1 mm depth) were maintained (Figure 8C). Similar intracellular inclusions as in the culture experiments could be observed in both UV-C exposed and positive control filaments (Figure 9E). Correspondingly, the analysis of elemental composition and structure rendered approximately round structures peaking in the elemental P, Ca, Mg, S, and sporadically K spectra (Figures 9A–D).

## Discussion

### Taxonomic Composition of Microbial Mat Layers

The Lagoa Vermelha microbial mat has been maintained for several years under controlled laboratory conditions to serve as a model ecosystem far from its place of origin (Vasconcelos et al., 2006, 2014; Vasconcelos and McKenzie, 2009). Since most natural communities change and adapt to laboratory conditions, it is difficult to extrapolate laboratory results to the field. However, here we show that several of the key functions (photosynthesis, presence of sulfur cycling taxa and calcification) remain intact with several of the natural key species performing these tasks (Supplementary Tables S3, S4).

The number of reads recovered by 16S rRNA amplicon sequencing using archaeal and bacterial primer sets revealed an inverse relationship in abundance between Archaea and Bacteria that, respectively, increase and decrease with depth (Figure 2). Although not quantitative, a potential higher archaeal abundance in the interior is consistent with studies on microbial mats in the natural stromatolite-forming Shark Bay area (Papineau et al., 2005; Wong et al., 2017). Also the overall microbial composition and the characteristic layering including a green photosynthetic layer, a white crystalline layer and a black sulfide rich layer are common for calcifying microbial mats such as from Shark Bay and Lagoa Vermelha (Vasconcelos et al., 2006). Hence, we consider the laboratory-incubated mats a suitable reference for natural stromatolite forming systems. The considerable incidence of Archaea in the bottom layer (Figure 2) is mostly attributed to the abundance of a novel group of Archaea that could only be assigned at the class level to Lokiarchaeia of the Asgard phylum using the SILVA version 132 as reference database. NCBI blast analysis returns a Methanothermobacter species (Euryarchaeota) as best blast hit albeit at only 78% sequence identity. We can rule out sequencing artifacts since many >99% sequence identity hits are found when compared to the nt database (Supplementary Table S3), with as best blast hits uncultivated species obtained from a molecular study of microbialites from hypersaline microbial mats. At this moment, we cannot give a conclusive identification, but members of the Asgard group, including Lokiarchaeia, have been observed before in stromatolites (Wong et al., 2018). Similarly, the archaeal OTUs annotated as Bathyarchaeia only have high sequence identity to cultivation independent obtained sequences from hypersaline environments and only a ∼80% identity with cultivated species. Little is known about this group of Archaea, but they may form a symbiotic association with Methanomicrobia with whom they often co-occur (Xiang et al., 2017). The genus *Methanohalophilus* within the order Methanosarcinales, is the most abundant archaeal genus in the top three layers and hints to an important contribution of potentially hydrogenotrophic methanogenesis in the mats. Hydrogenotrophic methanogenesis is considered an ancestral form of methane production (Bapteste et al., 2005; Wong et al., 2017; Lackner et al., 2018) and supports our interpretation that the Lagoa mat is a good analog of both modern and ancient stromatolites. The Thermoplasmata, represented by the Marine Benthic Group D and DHVEG-1 are found especially in the lower layers and may contribute to the sedimentary cycling of carbon, which might assign a key role of these organisms in lithifying mats (Zhou et al., 2019).

The overall composition is typical for microbial mats with a dominance of Proteobacteria, Bacteroidetes and Cyanobacteria, dependent on the sampling depth (Bolhuis and Stal, 2011). Primary production in the stromatolite type microbial mats is performed by oxygenic photosynthetic Cyanobacteria of the genus *Geitlerinema*, the dominant, filamentous cyanobacterium, *Dactylococcopsis*, a unicellular species and the filamentous genus of *Halomicronema* (Supplementary Table S4). Each of these salt tolerant cyanobacterial genera are frequently found in hypersaline environments (Oren, 2015) and stromatolites (Samylina and Zaytseva, 2019). Sulfur cycling is performed in the deepest layer by sulfur oxidizing bacteria (*Thiohalospira*) and sulfate reducing bacteria (order Desulfobacterales and Desulfovibrionales) (Supplementary Table S4). Sulfate reduction has been suggested to play a key role in the precipitation of carbonates in modern calcifying microbial mats, and recently a novel member of the *Desulfovibrionaceae* family has been linked to potential calcium carbonate deposition in a hypersaline environment (Spring et al., 2019).

The overall dominant genera are mainly anaerobic or microaerophilic heterotrophic bacteria, such as Balneolaceae_g, *Marinobacter* (facultative aerobe heterotrophs) and *Marinospirillum* (micro-aerophilic heterotroph) (Supplementary Table S4). Those genera may be involved in lamination formation requiring anoxic conditions and EPS nucleation sites (Vasconcelos et al., 2006).

Anaerobic, halophilic *Halanaerobium* taxa forming hydrogen and metabolizing C6 sugars, as well as *Spirochaeta* are potential fermenting bacteria. Fermentation can potentially counteract the calcification process, but may be prone to diel fluctuations (Dupraz et al., 2009). The presence of anaerobic phyla in the oxic top layers may be explained with sampling cross-overs or scattered anaerobic micro-zones (Wong et al., 2017). However, sulfate reducers, often considered as anaerobes, have also been found in (micro-)oxic regions of a mat suggesting active sulfur cycling within the upper layers (Minz et al., 1999a, b). The hypersaline nature of the microbial mat is reflected in the occurrence of halophilic species amongst the Archaea (*Halomarina* and *Halomicroarcula*) and halotolerant bacterial members (e.g., *Halanaerobium*, *Halomonas*) (Supplementary Table S4).

### Precipitation and Lithification of a Laboratory-Incubated Stromatolite

The laboratory-controlled stromatolite continues to accrete carbonate mainly via *in situ* precipitation, which is similar to Precambrian systems and their modern analogs such as alkaline lake and Lagoa Vermelha stromatolites (Kazmierczak and Kempe, 2006; Vasconcelos et al., 2006). Spadafora et al. (2010) analyzed the mesostructure of the specimen we study here, and disclosed an overall autochthonous peloidal matrix, while allochthonous granules only constitute a marginal percentage of the total volume. In general, Lagoa Vermelha stromatolites are laminated on a sub-mm scale and have been described as good textural analogs to Precambrian forms (Vasconcelos et al., 2006, 2014; Spadafora et al., 2010). The mineralogical character of the studied precipitates (Figure 3) is consistent with the results of earlier analyses of lithifying microbial mats in Lagoa Vermelha (van Lith et al., 2002; Moreira et al., 2004; Vasconcelos et al., 2006, 2014), despite the stromatolite’s transfer to a laboratory environment 15 years ago. A pronounced deviation from the natural system is the exclusive precipitation of magnesium calcite instead of additional dolomite. van Lith et al. (2002) disclose high salinity as a critical control on the formation of dolomite, which mainly occurs in early summer periods promoting evaporation in Lagoa Vermelha. The periodic fluctuation of water levels is not mimicked in the laboratory-incubated microbial mat. The hypersalinity may thus not be pronounced enough for dolomite precipitation on the stable laboratory-incubated stromatolite. The sediment presumably supplies a sufficient amount of Si to be sporadically trapped in the cyanobacterial EPS associated with magnesium calcite spheroids (Figure 3B).

The varying δ^13^C values (Table 1) throughout the carbonate stromatolite layers, which include primary precipitates (Top 0) and the amalgamated carbonate deposit (Top 1–Top 4) (Figure 1), indicate the contribution of different metabolic pathways to magnesium calcite deposition. Autotrophic carbon fixation features a strong preference for the lighter ^12^C, which manifests in the OM itself after isotopic fractionation, e.g., in the ribulose biphosphate carboxylase (RuBisCO) reaction of the C3 photosynthetic Calvin cycle prevalent in cyanobacteria (Schidlowski, 2001). This depletes the dissolved inorganic carbon (DIC) pool of ^12^C, thus increasing the ratio of heavy ^13^C and accounting for relatively positive δ^13^C values in locally formed inorganic material. Consequentially, values relatively negative compared to the equilibrium δ^13^C_DIC_ point to heterotrophic incorporation of OM in diagenetic processes (Brady et al., 2013). While methanotrophy produces an extremely negative δ^13^C, sulfate reduction leads to a considerable decrease in the ^13^C ratio of residual DIC as well. The layer of crystalline precipitates in the microbial mat features a relatively negative δ^13^C (Table 1), conversely to the positive values of carbonate mediated via photosynthesis. It is likely that diagenetic processing by aerobic heterotrophic bacteria consuming cyanobacterial necromass and EPS, as well as sulfate reduction in the anoxic zone of the microbial mat produces an increased ^12^C ratio in primary precipitates. Degradation of OM has been suggested to lead to formation of microbialites over time, while the upper mat layers presumably produce carbonates of globular morphology (Spring et al., 2019). Especially the significance of sulfate reducers in Lagoa Vermelha carbonate diagenesis has been shown before, since in addition to the consumption of organic compounds, this metabolic pathway produces alkalinity (Vasconcelos et al., 2006, 2014). Consequentially, the positive δ^13^C value of the most recently amalgamated calcite in the Top 1 upper part of the deposit, as well as the merely negative δ^13^C of the porous layer below indicate less prominent heterotrophic diagenesis, while the adjacent fine-grained layer and the very bottom of the stromatolite seem to incorporate a relatively higher ratio of metabolites enriched in ^12^C. Next to the consumption of autotrophic necromass, photosynthesis itself may mediate ^13^C enriched DIC in a CO_2_ limited system. The laboratory growth conditions simulate the increased salinity of Lagoa Vermelha and thus decrease solubility of CO_2_ (Weiss, 1974). It has been shown that the cyanobacterial response of concentrating inorganic HCO_3_^–^ can elevate ^13^C ratios in the OM, thus accounting for a DIC pool relatively more enriched in ^12^C (Sharkey and Berry, 1985; Fielding et al., 1998). Therefore, a combination of both heterotrophy and operating carbon concentrating mechanisms (CCM) may lead to precipitation and diagenesis of carbonate with a more negative δ^13^C than material mediated through regular C3 photosynthesis. The near zero δ^18^O values throughout the layers indicate an approximate equilibrium of precipitated and lithified carbonates with the aquatic environment, as well as a relatively similar isotopic composition of the Lagoa Vermelha natural seawater and the laboratory saline solution (including minor fluctuations) when comparing unconsolidated precipitates to the mature deposit.

### Growth and Carbonate Precipitation in *Geitlerinema*-Dominated Culture

The cyanobacterial enrichment identified *Geitlerinema* sp. as the main constituent. This species is also the most abundant cyanobacterium present in the top layer of the laboratory stromatolite (Supplementary Table S4) and has been observed in natural stromatolites (Samylina and Zaytseva, 2019), while Vasconcelos et al. (2006) found the genus *Microcoleus* (Oscillatoriales) to be one of the dominant taxa in a Lagoa Vermelha microbial mat. *Geitlerinema* sp. showed a strong tendency to assemble in dense associations (Supplementary Figure S3) protecting the microbial mat against mechanical forces in a natural ecosystem. While bioturbation of the sediment is mostly non-prevalent and higher eukaryotic organisms such as Vertebrates and Crustacea have merely been found in Lagoa Vermelha (Vasconcelos et al., 2006), carbonate environments commonly feature pulses of sedimentation due to episodically heavy, abiotic disruptions such as storms and floods (Schieber, 2007). Culture growth on agar indicates a general adaption to the periodically semi-arid conditions with direct air exposure common in Lagoa Vermelha, with the gelatinous agar itself simulating a mat-like substrate.

The mono-phototrophic community featured precipitation of magnesium calcite on agar plates (Figures 5, 6), but not in liquid medium. This suggests that a gel matrix favors mineral precipitation in the presence of *Geitlerinema* and that such media are good candidates to reproduce the substrate of natural microbial mats in general. Carvalho et al. (2018) studied a stromatolite from the same natural community as ours and disclosed a mean growth rate of 0.19 ± 0.03 mm/y. This indicates that the mat precipitates plus a section of the upper deposit layer of the stromatolite studied here have been formed in the laboratory and are especially valid to compare to the carbonates derived from cultures. Biominerals precipitated in those mono-phototrophic cultures are both chemically and morphologically similar to the ones precipitated in the natural microbial mat (Figures 3, 6). While it is possible that other relevant cyanobacterial species were outcompeted in the experiment, this suggests a significant contribution of *Geitlerinema* to primary carbonate mineral precipitation on the studied stromatolite at least. A prominent difference in chemical composition, however, is the overall lower magnesium content in culture precipitates. This phenomenon is most likely accounted for by a shortage of magnesium in the growth medium (Supplementary Table S1). Mg^2+^ ions are generally present in significantly hydrated form and react slower than Ca^2+^ ions, making them less bioavailable and presumably requiring even higher initial concentrations than introduced in order to balance the disparity.

The solid, nutrient-rich agar surface promotes biomineralization in particular, since it is not subjected to fluid dynamics and allows a locally permanent change of the chemical milieu in an area beyond the bacterial EPS layers. Photosynthetically induced ion gradients are visible on the agar as spots of darker discoloration in the process and facilitate a local concentration of Ca^2+^ and Mg^2+^, increasing both biomineral saturation state and pH (Kaźmierczak et al., 2015). An abiotic factor further assisting ion concentration and thus calcite formation is evaporation. The overall higher culture volume renders this parameter less significant in liquid medium, but cannot be neglected on agar plates subjected to high temperatures for prolonged periods of time. Evaporation entails the transition of the lighter stable oxygen isotope ^16^O to the atmosphere, shifting the isotopic value of the remaining DIC pool to comparably positive δ^18^O values. Since the δ^18^O value of the laboratory distilled water is −8.47‰ SMOW, the average isotopic ratio of 0.39‰ VPDB in culture-precipitated carbonates may indicate significant evaporation in the solid growth medium, which could also be observed in the gradual desiccation of agar plates over the course of the experiment, despite their transfer to plastic bags. However, carbonate precipitation was exclusively observed on top of cyanobacterial mats and in no more than half of agar cultures kept under identical conditions, rendering an abiotic precipitation unlikely and indicating cyanobacterial mediation. Interestingly, the δ^18^O of morphologically similar carbonate precipitates embedded in the aquarium-kept stromatolite microbial mat is equally positive (Table 1).

In contrast, the δ^13^C of −12.26 in carbonates derived from culture experiments, is extremely negative compared to the values derived from the carbonate layers of the stromatolite itself (Table 1) and simultaneously seems to contradict an abiotic and photosynthetic origin. The carbonate crusts were sampled in careful avoidance of OM, which could explain the distinctly negative value. However, the growth medium contains an extremely elevated carbonate concentration (Supplementary Table S1). While atmospheric CO_2_ consists to approximately 99% of ^12^C, inorganic (bi-) carbonate features a generally higher ^13^C ratio (Sharkey and Berry, 1985). Similar to biomineralization and diagenetic processes on the stromatolite, a greater tendency to take up the heavier isotope from the abundant bicarbonate during C fixation may explain a lower isotopic discrimination than expected and thus exceedingly negative results in the DIC pool. The RuBisCO enzyme adds fixed CO_2_ to ribulose-1,5-bisphosphate, eventually mediating the formation of carbohydrates during the photosynthetic dark reaction and resulting in a prominent isotopic fractionation (Schidlowski, 2001). Many cyanobacteria including *Geitlerinema* sp. (Batchu et al., 2019), however, are able to simultaneously take up inorganic HCO_3_^–^, which is assimilated in the cytoplasm and converted into CO_2_ in the carboxysome. Such CCMs are not only activated in hypersaline or hot environments, but especially in alkaline conditions when CO_3_^2–^ dominates and allow the cells to survive CO_2_ limitation (Kamennaya et al., 2012).

The conversion of HCO_3_^–^ into CO_2_ for carboxylation consumes H^+^ and contributes to the cell surface alkalization, where Ca^2+^ and Mg^2+^ ions are bound by the anionic EPS and precipitated with CO_3_^2–^ generated from HCO_3_^–^ as a result of the increased pH. Simultaneously, the alkaline milieu shifts the equilibrium of the bicarbonate buffer system to the right, further increasing HCO_3_^–^ and CO_3_^2–^ concentrations (Kamennaya et al., 2012). In addition to the higher ionic bioavailability, the swift precipitation of calcium carbonate may also be explained by an additional accumulation of calcium cations via cellular Ca^2+^/H^+^ antiporters at this point, requiring even higher initial concentrations for comparable Mg^2+^ biomineralization. The nucleating carbonate crystals can be observed adhered to the EPS as globules typical for this phase (Smeets et al., 2015) (Figures 6B,C). Spheroids amalgamate in later stages and form calcite in different textures giving distinct electron diffraction signals but featuring an overall rather poorly crystallized character.

The irregular crystalline diffraction pattern (Figure 6D) distinguishes the comparably calcium-rich intracellular inclusions of cells cultured in liquid medium, which gave no measurable signal at all (Figure 7B). While it is possible that the ED detector was blocked by the considerable layer of OM or indicated intracellular calcium carbonate precipitates of a very low degree of crystallinity, the high phosphorous peak cannot be explained by the general P content in the cell since it is considerably concentrated in the inclusions (Figure 7B). The overall EDS spectrum (Figure 9C) is typical for polyphosphate (PolyP) granules, which are stored in the form of magnesium/calcium salts and formed by cyanobacterial taxa (Benzerara et al., 2014; Feng et al., 2018). Those P storage units have been described as “bioenergy fossils” and were probably already present in the prebiotic era (Achbergerová and Nahálka, 2011). Adopting several cellular functions such as energy supply and regulation of metabolic processes, PolyP granules can be interpreted as a stress response to starvation (Achbergerová and Nahálka, 2011; Racki et al., 2017), which indicates nutrient limitation in the aged liquid medium. The PolyP inclusions may represent another stable adaption of the cultured *Geitlerinema* to the dynamic conditions in Lagoa Vermelha, facilitating long-term mat survival and stromatolite growth. Concurrently, morphological, chemical and isotopic characteristics of the extracellular carbonate deposits document the mineralization of carbonates similar to the primary precipitates of the laboratory-incubated stromatolite and in the mono-phototrophic culture and represent an important precondition for the interpretation of *in situ* precipitated fossil stromatolites.

### UV-C Resilience of *Geitlerinema*-Dominated Cultures

Another crucial factor for the evaluation of past terrestrial and a potential Martian atmospheric oxygenation is cyanobacterial resilience toward UV-C radiation. The high energy of UV photons is illustrated by the precipitation of iron- and manganese-rich flakes from liquid ASN-III medium in a presumably UV-C catalyzed abiotic reaction, since it is apparent in the absence of bacterial cells as well but not in cultures shielded from UVR (Anbar and Holland, 1992) (Figures 8B, 9A). Interestingly, the growth medium contains a considerable amount of Fe, but only minuscule traces of Mn introduced by the trace metal mix (Supplementary Table S1), with no corresponding materials present in tools or vessels. A possible source of contamination may be the use of not entirely demineralized water containing residues of metal ions. Abiotic formation of putative manganese oxide via shortwave solar radiation could be relevant to early Earth conditions and potentially to conditions on Mars, since it is currently believed that a combination of both oxygen (or oxygen radicals) and microbial activity is required to overcome respective kinetic barriers (Spiro et al., 2010). The readily adsorbance of metal ions by bacteriogenic MnO_2_ might render its presence especially meaningful for carbonate depositing systems, but further analysis of the composition of manganese-rich flakes precipitated by UV-C irradiation would be needed to gain insight into this process.

The highly energetic effect of the shortwave UV radiation did not allow bacterial growth on agar, as opposed to the liquid medium where cultivation of few mats could be observed (Figure 8A). In accordance with both cultivation experiment results and the significantly shortened time frame, this involved lack of extracellular precipitation but formation or maintenance of PolyP granules presumably facilitating stress acclimation (Figure 9).

A cyanobacterial key defense mechanism against UVR is the behavioral avoidance of damaging solar radiation concentrations. *Geitlerinema* species are capable of gliding motility and Oscillatoriales have been reported to migrate within microbial mats depending on the spectrum of incident wavelengths (Ramsing and Prufert-Bebout, 1994; Johansen et al., 2017). The natural mat system represents an ideal refuge, in which UVR tolerant species build a protective top layer for other, motile phototrophs and the deeper community (Quesada and Vincent, 1997). In the experimental set-up, the UV-C blocking microscope slide provides an abiotic type of shelter. In natural Early Earth and Mars analogs, porous rocks (Albanese et al., 2020), ice and dust (Rontó et al., 2003) might take a similar role. *Geitlerinema* filaments exclusively settled on the bottom side of the glass, as opposed to the positive control where mats formed on top of the slide (Figure 8B), closer to the PAR source. Cells apparently reached the refuge in two liquid cultures only (Figure 8A), potentially indicating an adverse distribution upon inoculation combined with an innate or UVR-induced, limited swimming ability. Indeed, it has been shown that long-term UVR exposure impairs gliding motility in filamentous cyanobacteria (Donkor and Haeder, 1991). At the same time, growth in liquid positive controls was even more limited, a phenomenon that may be explained by pronounced PAR absorption of the liquid medium. While solid medium cultures proliferated extensively in positive controls (Figure 8A), the agar surface did not provide any shelter from harmful irradiation. Similar to burial in sediments or mats, however, its subsurface evidently offers sufficient protection for the maintenance of pre-established filaments in a very shallow depth already (Figure 8C). This suggests that the cultures are not UV-C resistant enough to emerge primary mats from single cells in surface environments operating energy-intensive repair or blocking mechanisms, and that single cells may not be able to bury themselves in firm substrate and/or UVR environments. However, our experimental set-up does not account for an Archean “faint young sun,” which featured a luminosity reduced up to 70% of present solar radiation (Kasting, 2010). In Early Earth systems, the lower brightness of the sun might thus have facilitated the establishment of comparable photosynthesizing mats regardless. In any case, the cultivate features sufficient resilience to add to the community when sheltered by abiotic refuges or, accordingly, sessile cyanobacteria producing UV blocking agents. In a natural and non-PAR-permeable system, this is additionally dependent on unconstrained motility of phototactic mature filaments to simultaneously facilitate photosynthetic activity and avoidance of harmful solar radiation doses. These conclusions are not only relevant for astrobiological considerations, but also for modern terrestrial environments that promote cyanobacterial biomineralization and the formation of stromatolites subjected to elevated UVR intensities in high latitudes (Phoenix et al., 2006; Farías et al., 2013).

Our results suggest that modern *Geitlerinema* taxa are dependent on shelters enabling an escape from lethal doses of short-wave UVR. Even though this does not allow hard conclusions on the presence or absence of cyanobacteria in the Archean, it underlines the relevance of diversification in putative cyanobacterial mats during the Earth’s oxygenation, which provided biotic refuges for motile phototactic cyanobacteria and eventually promoted a global UV-C sanctuary for the evolution of all present domains of life. Based on our observations, we propose that cyanobacteria-derived carbonate deposits are hotspots for traces of aerobic life and support further research on the performance of cyanobacteria under early Earth and Martian conditions. The consideration of biomineralizing anoxygenic photosynthetic taxa such as purple sulfur bacteria (Warthmann et al., 2011) may be an especially valuable addition to further attempts in shedding light on the evolution of aerobic life.

## Conclusion

A metabolically diverse community accounts for precipitation and diagenesis of magnesium calcite on a laboratory-controlled stromatolite. Although not the overall dominant species, *Geitlerinema* sp. is the major cyanobacterial primary producer in this system and responsible for the oxygenation of the top layer. The *Geitlerinema* enrichment was shown to facilitate carbonate precipitation and was able to endure long-term exposure to highly energetic UV-C radiation. The unexpected large number of yet uncultivated Archaea related to the Asgard group may allow us to study this newly identified phylum and facilitate their cultivation and investigation, while the abiotic precipitation of putative manganese oxides via high-energy solar radiation might be of interest regarding the geochemical cycles of pre-GOE systems. Finally, this study will further contribute to our search for putative extraterrestrial live and especially fossilized biota as well as potential oxygenation of the presently UV-C permeable atmosphere on Mars.

## Data Availability Statement

The raw data supporting the conclusions of this article will be made available by the authors, without undue reservation, to any qualified researcher. The sequencing data has been submitted to the NCBI-SRA database under BioProject ID PRJNA610984.

## Author Contributions

MS-R performed conception and design of the study, assessed results and advised in practical execution, and manuscript writing. RP contributed to study design, conducted practical work, and assessed results as an internship part of the MSc track Freshwater and Marine Biology, University of Amsterdam, and also wrote the draft of the manuscript. HB performed both practical and analytical taxonomic assessment and contributed substantially to the writing and development of the manuscript. GM contributed as adviser, reviser and to the development of the manuscript. All authors contributed to manuscript revision, read and approved the submitted version.

## Conflict of Interest

The authors declare that the research was conducted in the absence of any commercial or financial relationships that could be construed as a potential conflict of interest.
