# Supplementary material for: Stromatolites as Biosignatures of Atmospheric Oxygenation: Carbonate Biomineralization and UV-C Resilience in a Geitlerinema sp. - Dominated Culture
Source: Front Microbiol. 2020 May 19;11:948. doi: 10.3389/fmicb.2020.00948 (PMC7248245; doi:10.3389/fmicb.2020.00948)
Supplement: Supplementary file 1 [file Data_Sheet_1.PDF]

Supplementary Material

Stromatolites as biosignatures of atmospheric oxygenation: Carbonate biomineralization and UV-C resilience in a *Geitlerinema* sp. - dominated culture

Rabja Maria Popall, Henk Bolhuis, Gerard Muyzer, Mónica Sánchez-Román*

*** Correspondence:** Mónica Sánchez-Román - email: m.sanchezroman@vu.nl

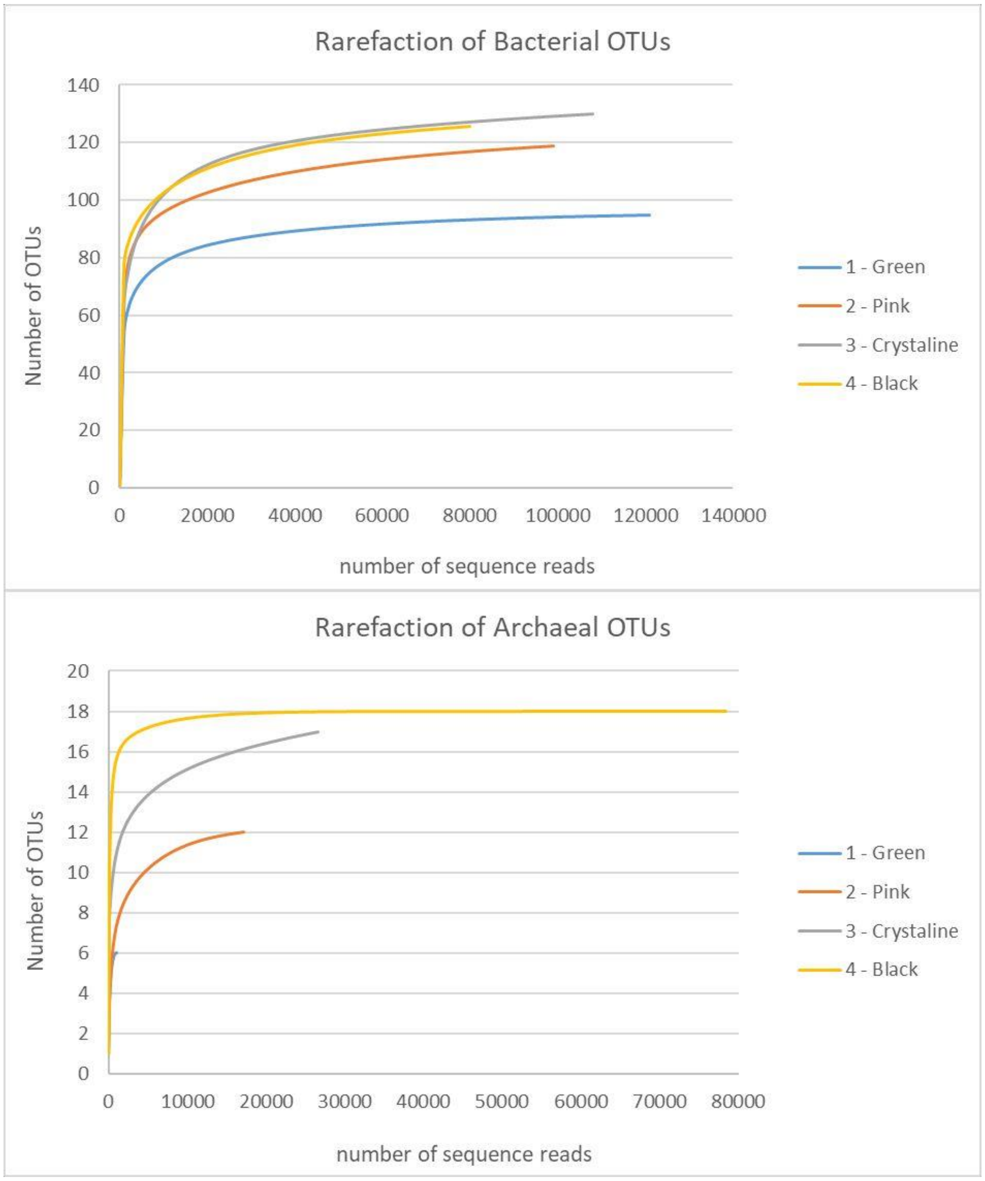

Supplementary Figure S1: Rarefaction curves of bacterial and archaeal OTUs

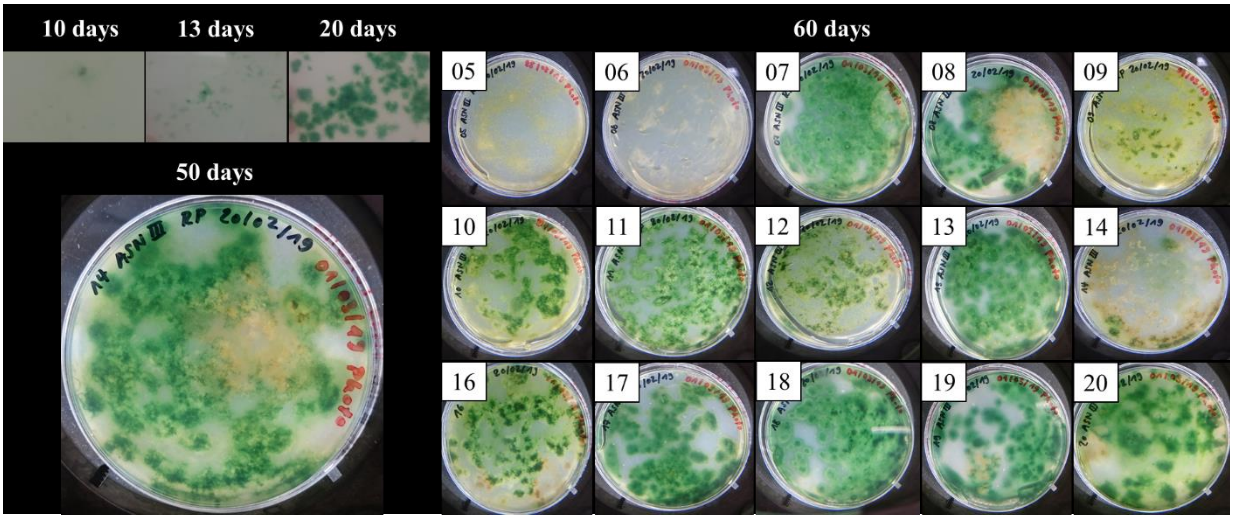

Supplementary Figure S2: Culture growth on solid ASN-III medium over time. After 60 days, the filamentous surface cover started to recess. Plate 06 had been overgrown by light-green bacterial mats prior to being sampled for taxonomic identification. Additionally, plate 05 and 09 feature light-green overgrowth as opposed to the remaining 13 dishes. Plate 15 was overgrown by dark-green filaments and given to NIOZ after sampling for isolate identification, hence it is missing in the scheme. Plate numbers 01-04 were not assigned to this experiment. Plates 07, 08, 14, 16, 19 and 20 feature browning after 60 days.

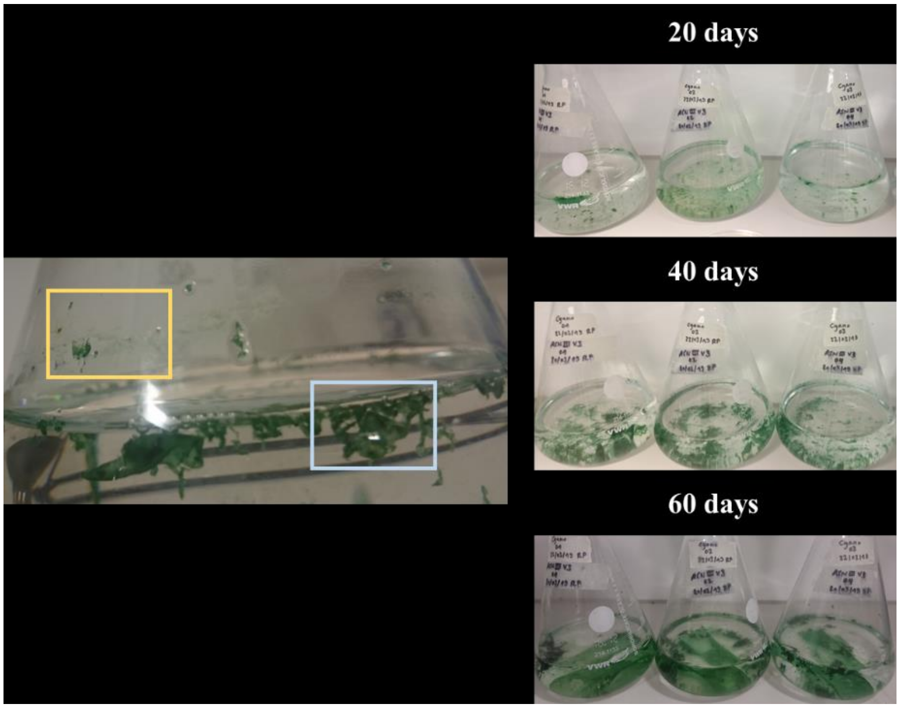

Supplementary Figure S3: Culture growth in liquid ASN-III medium over time. Cultures settling on the glass surface are marked in yellow and swimming mats in blue.

Supplementary Table S1. ASN-III media compositions in culture (C) and UVR (U) experiments, as well as solid (S) and liquid (L) and A-5 trace metal mix after Rippka et al. (1979). Modifications to the original recipe are highlighted in light blue. The pH of culture experiment medium was adjusted to ~7.5 with 1M acetic acid prior to autoclaving if applicable. The bicarbonate for UVR experiments was filter-sterilized. $CaCl_2$ was autoclaved separately to avoid abiotic precipitation at high temperatures.

Culture experiments:

**ASN-III-CS medium**	
**Amount /liter**	**Ingredient**
1,000ml	Deionized water
35g	NaCl
3.5g	$MgSO_4 * 7H_2O$
8.0g	$MgCl_2 * 6H_2O$
1g	$CaCl_2 * 2H_2O$
0.5g	KCl
0.75g	$NaNO_3$
0.015g	K_2HPO_4
1.0g	Na_2CO_3
0.0005g	EDTA
0.0033g	Citric acid monohydrate
0.003g	Ferric ammonium citrate
1ml	Trace metal mix A-5
20g	Agar

**ASN-III-CL medium**	
**Amount /liter**	**Ingredient**
1,000ml	Deionized water
70g	NaCl
3.5g	$MgSO_4 * 7H_2O$
8.0g	$MgCl_2 * 6H_2O$
1g	$CaCl_2 * 2H_2O$
0.5g	KCl
0.75g	$NaNO_3$
0.015g	K_2HPO_4
0.25g	$NaHCO_3$
0.0005g	EDTA
0.0033g	Citric acid monohydrate
0.003g	Ferric ammonium citrate
1ml	Trace metal mix A-5
-	-

UVR experiments:

**ASN-III-US medium**	
**Amount /liter**	**Ingredient**
1,000ml	Deionized water
35g	NaCl
3.5g	$MgSO_4 * 7H_2O$
8.0g	$MgCl_2 * 6H_2O$
1g	$CaCl_2 * 2H_2O$
0.5g	KCl
0.75g	$NaNO_3$
0.015g	K_2HPO_4
0.07g	$NaHCO_3$
0.0005g	EDTA
0.0033g	Citric acid monohydrate
0.003g	Ferric ammonium citrate
1ml	Trace metal mix A-5
20g	Agar

**ASN-III-UL medium**	
**Amount /liter**	**Ingredient**
1,000ml	Deionized water
35g	NaCl
3.5g	$MgSO_4 * 7H_2O$
8.0g	$MgCl_2 * 6H_2O$
1g	$CaCl_2 * 2H_2O$
0.5g	KCl
0.75g	$NaNO_3$
0.015g	K_2HPO_4
0.07g	$NaHCO_3$
0.0005g	EDTA
0.0033g	Citric acid monohydrate
0.003g	Ferric ammonium citrate
1ml	Trace metal mix A-5
-	-

**Trace metal mix A-5**	
**Amount /liter**	**Ingredient**
1,000ml	Deionized water
2.86g	H_3BO_3
1.32g	$MnCl_2$ * $1H_2O$
0.222g	$ZnSO_4$ * $7H_20$
0.039g	Na_2MoO4 *$2H_2O$
0.079g	$CuSO_4$ * $5H_2O$
0.0494g	$Co(NO_3)_2$ * $6H_2O$

Rippka, R., Deruelles, J., Waterbury, J. B., Herdman, M., and Stanier, R. Y. (1979). Generic Assignments, Strain Histories and Properties of Pure Cultures of Cyanobacteria. *Journal of General Microbiology* III, 1–61.

Supplementary Table S2. Diversity and Richness indices of the 4 mat layers.

	Bacteria				Archaea			
Layer	reads	Shannon_H	Evenness	Chao-1	reads	Shannon_H	Evenness	Chao-1
1 - Green	122208	2,89	0,19	96	1131	0.37	0.24	6
2 - Pink	100217	2,95	0,16	125	17383	0.19	0.10	12
3 - Crystalline	109666	2,82	0,13	135	26783	1.03	0.16	17.5
4 - Black	81969	3,22	0,20	135	78595	1.07	0.16	18

Supplementary Table S3. Percent identity of archaeal and bacterial OTUs to best blast hits against the NCBI nt database (% nt) and the 16S ribosomal RNA (Bacteria and Archaea) type strain database (% type strains)

Archaeal OTUs			Bacterial OTUs		
OUT_ID	% nt	% type strains	OUT_ID	% nt	% type strains
OTU_29	99.5	78.8	OTU_18	99.4	99.4
OTU_30	99.5	78.7	OTU_19	96.6	95.7
OTU_25	97.8	79.2	OTU_80	99.6	89.9
OTU_26	98.8	82.3	OTU_81	98.7	97.8
OTU_28	93.7	79.9	OTU_20	98.1	96.6
OTU_13	96.7	95.2	OTU_23	99.4	98.9
OTU_14	99.5	98.6	OTU_43	95.3	94.7
OTU_15	99.0	95.5	OTU_22	99.1	89.1
OTU_16	99.0	97.6	OTU_47	96.4	96.4
OTU_17	99.5	99.3	OTU_111	99.8	92.4
OTU_18	99.5	80.9	OTU_21	99.4	98.5
OTU_19	99.0	82.1	OTU_155	93.9	84.2
OTU_20	96.9	82.8	OTU_24	99.4	98.7
OTU_21	99.3	82.8	OTU_26	98.5	96.6
OTU_22	97.3	82.7	OTU_92	95.0	94.0
OTU_23	99.5	82.1	OTU_136	99.6	98.0
OTU_24	96.4	82.3	OTU_77	98.7	83.4
OTU_27	98.8	91.8	OTU_25	99.6	89.6

For bacteria, only the most abundant OTUs are depicted in this table.

Supplementary Table S4. Percent relative abundance the 20 most abundant bacterial genera per layer. Taxa without cultured representatives and thus no given names are identified with _f and _*g* prefixes.

**1 – Green**		**2 – Pink**		**3 – Crystalline**		**4 - Black**	
**Genus**	**%**	**Genus**	**%**	**Genus**	**%**	**Genus**	**%**
Balneolaceae_*g*	34.02	*Marinobacter*	35.00	*Marinobacter*	37.48	*Marinospirillum*	29.75
Spirochaeta 2	21.34	*Marinospirillum*	20.93	*Marinospirillum*	24.65	*Marinobacter*	17.93
Marinobacter	15.65	Balneolaceae_*g*	11.30	Spirochaeta 2	5.23	*Halanaerobium*	14.43
Marinospirillum	12.09	Spirochaeta 2	6.54	*Halanaerobium*	3.65	Spirochaeta 2	7.49
Geitlerinema	6.47	Phycisphaeraceae_*g*	5.85	*Marinimicrobium*	3.50	Oligoflexales_f_*g*	4.26
Halanaerobium	1.54	*Halanaerobium*	5.07	Fodinicurvataceae_*g*	2.95	Marinilabiliaceae_*g*	3.24
Marinimicrobium	1.15	*Geitlerinema*	1.86	Phycisphaeraceae_*g*	2.91	*Sediminispirochaeta*	3.14
Salinarimonas	0.98	*Alkalispirochaeta*	1.48	Balneolaceae_*g*	2.76	Clostridiales_f_*g*	2.32
Gammaproteobacteria_f_*g*2	0.81	*Sediminispirochaeta*	1.11	*Alkalispirochaeta*	2.40	*Desulfosalsimonas*	2.12
Halomicronema	0.80	Gammaprot._KI89A clade_f_*g*	1.08	*Pelagibius*	2.15	Desulfobacteraceae_*g*	1.63
Dichotomicrobium	0.63	*Oceanicaulis*	0.83	*Dichotomicrobium*	1.73	Balneolaceae_*g*	1.42
Fodinicurvataceae_*g*	0.56	Gammaproteobacterium_f_*g*	0.83	*Sediminispirochaeta*	1.49	Atribacteria_f_*g*	1.11
Nodosilineaceae_*g*	0.43	*Dichotomicrobium*	0.66	*Lewinella*	1.21	Bacteroidetes_f_*g*	1.10
Anaerophaga	0.42	Nitrosococcaceae_*g*	0.59	Planctomycete_f_*g*	1.18	Anaerolineae_SBR1031_f_*g*	0.98
Gammaproteobacterium_f_*g*	0.41	Rhizobiaceae_*g*	0.53	Gammaprot._KI89A clade_f_*g*	1.15	Phycisphaerales_f_*g*	0.86
Thalassobaculales_f_*g*	0.28	Fodinicurvataceae_*g*	0.51	Rhizobiaceae_*g*	0.46	*Alkalispirochaeta*	0.74
Ruegeria	0.27	*Anaerophaga*	0.48	Parvibaculaceae_*g*	0.43	Candidatus Marispirochaeta	0.68
Dactylococcopsis	0.25	Halobacteroidaceae_*g*	0.48	*Orenia*	0.39	*Desulfovibrio*	0.64
Saprospiraceae_*g*	0.23	*Wenzhouxiangella*	0.40	*Salinarimonas*	0.39	*Anaerophaga*	0.57
Nitrosococcaceae_*g*	0.22	*Ruegeria*	0.39	*Oceanicaulis*	0.35	Syntrophomonadaceae_*g*	0.52

Supplementary Table S5. Bacterial genera dominant in only one of the 4 microbial mat layers (indicated by grey shading).

	**1 - Green**	**2 - Pink**	**3 - Crystalline**	**4 - Black**
Oligoflexales_f_*g*	0	0.004	0.011	4.262
Clostridiales_f_*g*	0	0.001	0.001	2.317
Desulfobacteraceae_*g*	0	0	0.005	1.626
Phycisphaerales_f_*g*	0	0.003	0.006	0.856
Candidatus Marispirochaeta_*g*	0	0.004	0.005	0.682
Aerophobetes_f_*g*	0	0	0	0.434
Halocella	0	0	0	0.299
Bacteroidetes vadinHA17_*g*	0	0	0	0.278
Aminicenantales_f_*g*	0	0	0.001	0.271
Babeliales_f_*g*	0	0	0	0.261
Pelagibius	0.003	0.034	2.152	0.002
Defluviicoccus	0.002	0.256	0.001	0
Cryomorpha	0.013	0	0	0
